# Sleep Treatment Education Program for Young Adult Cancer Survivors (STEP-YA): Protocol for an Efficacy Trial

**DOI:** 10.2196/52315

**Published:** 2023-11-29

**Authors:** Alexis L Michaud, Briana Bice, Eva Miklos, Katherine McCormick, Cheryl Medeiros-Nancarrow, Eric S Zhou, Christopher J Recklitis

**Affiliations:** 1 Perini Family Survivors' Center Dana Farber Cancer Institute Boston, MA United States; 2 Department of Psychology Suffolk University Boston, MA United States; 3 Harvard Medical School Boston, MA United States

**Keywords:** insomnia, cancer survivors, young adults, protocol, coaching, mood

## Abstract

**Background:**

Young adult cancer survivors (YACS) are at elevated risk for chronic insomnia, even years after completing treatment. In addition to potential health consequences, insomnia can interrupt social, educational, and vocational development just as they are trying to “make up” for time lost to cancer. Cognitive behavioral therapy for insomnia (CBTI) is recommended as first-line treatment for insomnia but remains largely unavailable to YACS due to several barriers (ie, shortage of trained providers, geographic limitations, financial limitations). Traditional CBTI has not been adapted to meet YACS’ unique developmental and circadian challenges. To improve availability of effective behavioral insomnia treatment for this population, we developed the Sleep Treatment Education Program for Young Adult Cancer Survivors (STEP-YA), a low-intensity educational intervention delivered virtually online.

**Objective:**

In this phase 2 “proof of concept” trial, primary aims are to test the efficacy of STEP-YA to improve insomnia symptoms and mood in YACS and assess the utility of individualized coaching to improve treatment effects. A secondary aim will explore participant variables associated with clinically significant response to STEP-YA.

**Methods:**

This 2-arm randomized prospective trial will enroll 74 off-treatment YACS aged 20 years to 39 years with clinically significant insomnia. Each participant completes the STEP-YA intervention in a 1-on-1 synchronous online session led by a trained interventionist following a structured outline. The 90-minute intervention presents educational information on the development of insomnia after cancer and offers specific suggestions for improving insomnia symptoms. During the session, participants review the suggestions and develop a personalized sleep action plan for implementing them. After the session, participants are randomized to either the coaching condition, in which they receive 2 telephone coaching sessions, or the no-coaching condition, which offers no subsequent coaching. The Insomnia Severity Index (ISI) and the Profile of Mood States: Short Form (POMS-SF) are assessed at baseline and 4 and 8 weeks postintervention.

**Results:**

Enrollment began in November 2022, with 28 participants currently enrolled. We anticipate recruitment will be completed in 2024. The primary endpoint is a change in ISI score from baseline to 8 weeks postintervention. The secondary endpoint is change in mood symptoms (POMS-SF) from baseline to 8 weeks postintervention. Change scores will be treated as continuous variables. Primary analyses will use ANOVA methods. A within-subjects analysis will examine if the STEP-YA intervention is associated with significant changes in insomnia and mood over time. A 2-way ANOVA will be used to evaluate the utility of coaching sessions to improve treatment effects.

**Conclusions:**

Chronic insomnia has significant negative effects on YACS’ medical, educational, and psychological functioning. STEP-YA aims to address their needs; study results will determine if the intervention warrants future effectiveness and dissemination studies and if individualized coaching is necessary for adequate treatment response.

**Trial Registration:**

ClinicalTrials.gov NCT05358951: https://clinicaltrials.gov/study/NCT05358951

**International Registered Report Identifier (IRRID):**

DERR1-10.2196/52315

## Introduction

### Background

Because cancer disrupts their physical, emotional, and social development during a critical period in their life, young adult cancer survivors (YACS) are particularly vulnerable to treatment late effects, including insomnia [1-8]. Insomnia is commonly mistakenly viewed by patients and providers “as a temporary reaction to the cancer diagnosis or treatment” [9], with few long-term consequences. However, insomnia frequently develops into a debilitating chronic condition. As many as 1 in 4 YACS experience significant insomnia many years after cancer treatment has ended [5-7,10]. Chronic insomnia is associated with significant health problems in the general population, including heart disease, obesity, hypertension, diabetes, depression, and anxiety [11-20]. Because of their medical history, YACS are already vulnerable to many of these health conditions, making access to effective insomnia treatment critically important to their overall health [21-23].

Cognitive behavioral therapy for insomnia (CBTI) is a well-established and empirically supported treatment. CBTI is the recommended first-line treatment by the American Academy of Sleep Medicine and American Colleges of Physicians for insomnia disorder in adults [24,25]. Multiple randomized trials have demonstrated CBTI is the most effective long-term insomnia treatment, even compared with pharmacotherapy [26-28]. A review of CBTI for cancer survivors similarly concluded it “provides significant, lasting improvement” [29].

Unfortunately, CBTI is largely unavailable to the growing population of YACS who need it [30,31]. To make CBTI widely available to YACS, several barriers need to be resolved, including the shortage of trained CBTI providers [32,33], the patient burden of standard CBTI regimens [34-36], and the lack of CBTI interventions specifically targeting the needs of YACS. Moreover, as YACS’ access to effective insomnia care is limited by geography and other factors [30,31,33,37-39], it is imperative CBTI be made more accessible to them in their communities.

To address these challenges and deliver effective insomnia treatment to YACS, we developed the Sleep Treatment Education Program for Young Adult Cancer Survivors (STEP-YA). STEP-YA is a brief, low-intensity intervention presenting core elements of CBTI in an online educational format. Delivered in a 90-minute, 1-on-1, instructor-led session, STEP-YA educates YACS about causes of insomnia after cancer, including developmental, social, and health factors affecting YACS, and introduces them to CBTI principles and methods. During the synchronous (live) online session, survivors review their current sleep habits and consider alternative strategies consistent with CBTI recommendations. The standard elements of CBTI are presented in STEP-YA but with modifications previously shown to maintain efficacy while reducing treatment demands [40]. Additionally, information particularly salient to the experience of YACS was added, including information about how cancer diagnosis and treatment impact sleep and how to deal with young adult stressors (ie, fear of missing out, disconnecting). Following a self-management approach, the instructor provides guided behavioral planning, encouraging survivors to develop an individualized sleep action plan they will implement after the session.

### Objectives

The STEP-YA intervention is designed to address barriers to insomnia treatment for YACS because it is brief, low-cost, low-burden, and targets their specific needs and experiences. Delivered virtually, STEP-YA is available to YACS when and where they need it, without requiring travel to a specialized insomnia treatment site. As a self-management intervention, STEP-YA supports YACS’ autonomy by helping them make informed choices to manage their own insomnia [41], without taking the prescriptive stance commonly found in behavioral therapies. The efficacy of STEP-YA is currently being evaluated in a clinical trial described here. The primary aims of the study are to (1) test the hypothesis that STEP-YA improves insomnia symptoms (primary outcome) and mood (secondary outcome) in YACS and (2) determine if improvement in these outcomes is superior in the coaching condition compared with the noncoaching condition. A secondary aim of the study is to explore participant variables associated with clinically significant response to the STEP-YA intervention.

## Methods

### Study Design

Guided by the Obesity-Related Behavioral Intervention Trials (ORBIT) model for development and early testing of behavioral interventions [42-44], the STEP-YA intervention is being evaluated in an early-stage (phase 2A) randomized clinical trial. Participants are randomized (1:1) to receive the STEP-YA intervention either (1) alone (noncoaching condition) or (2) with the addition of 2 remote coaching sessions (coaching condition). STEP-YA sessions are delivered online via live videoconference. Outcomes are assessed at baseline and again 4 weeks and 8 weeks postbaseline. Participants are off-treatment YACS recruited from treatment centers, advocacy groups, and social media, and will complete all study activities remotely.

### Ethics Approval

Prior to study activation and participant enrollment, the study protocol was approved by the Dana-Farber/Harvard Cancer Center Institutional Review Board (Protocol 21-613).

### Participants

Participants will be 74 off-treatment YACS aged 20 years to 39 years. To be eligible for enrollment, YACS were diagnosed with cancer (excluding nonmelanoma skin cancer) at least 1 year prior, have received no cancer therapy (excluding chemoprevention) in the prior 4 months, and have no additional cancer therapy planned. Additionally, participants must be able to read and write in English and have significant insomnia as evidenced by an Insomnia Severity Index (ISI) ≥12 [45]. YACS who have specific conditions, medications, or past insomnia treatments that may affect their sleep or response to STEP-YA are excluded; these inclusion and exclusion criteria are detailed in Table 1.

**Table 1:** Study inclusion and exclusion criteria.

Category		Criteria	Rationale
**Inclusion criteria**			
	General	Age 20 years to 39 years Able to read and write in English	Enroll off-treatment YACS^a^ able to participate in the intervention currently available only in English
	Health issues	History of a cancer diagnosis (except nonmelanoma skin cancer) ≥1 year prior No active cancer therapy (excluding chemoprevention) in the past 4 months and no further therapy planned	Enroll off-treatment YACS able to participate in the intervention currently available only in English
	Sleep problems	Significant insomnia, as evidenced by an Insomnia Severity Index (ISI) score ≥12	To test this intervention with individuals with clinically significant insomnia
**Exclusion criteria**			
	General	Any impairment (hearing, visual, cognitive) that interferes with the ability to complete all study measures independently	Exclude individuals not able to independently complete the study procedures
	Health issues	Diagnosed with bipolar disorder Report being diagnosed with a seizure disorder or having experienced a seizure in the past 12 months	Exclude individuals for whom behavioral treatment for insomnia may require modification due to medical conditions
	Sleep problems	Intention to adjust (decrease or increase) use of any prescribed or over-the-counter medications taken to decrease insomnia during the study period Survivors who report being diagnosed with sleep apnea who are not receiving recommended medical treatment for their sleep apnea Survivors who report suspected sleep apnea who have not completed an evaluation by a sleep specialist Survivors who report their usual bedtime is not between 5 PM and 5 AM Employment that involves irregular sleep patterns, such as shift work or frequent long-distance travel across time zones, or employment in a position that could impact public safety	Exclude individuals whose sleep symptoms may require significant modifications in behavioral treatment for their insomnia
	Prior insomnia treatment	Prior participation in a research study that provided an educational or behavioral intervention for insomnia Prior participation in a behavioral treatment or patient education program for insomnia delivered at the study site Participation in behavioral or educational interventions for insomnia in the 2 years prior to enrollment (including synchronous and asynchronous online insomnia programs)	Enroll only participants naïve to the insomnia educational programs and behavioral treatments

^a^YACS: young adult cancer survivors.

### Recruitment

Recruitment materials and procedures are designed to allow YACS to self-refer to the study. These materials include a brief informational video, study information sheet, outreach letters and emails, informational postcards, and brief study descriptions suitable for posting on patient-facing materials distributed by patient support and advocacy groups. All materials provide phone, email, and web contact information for YACS interested in learning more about the study. Examples of these can be seen on the study website [46].

To distribute these materials to potentially eligible YACS, several methods are used. At our cancer center, recruitment postcards are placed in patient waiting areas, and study information appears on hospital screens presenting information and notices to patients. Clinical providers are informed of the study and given recruitment information and materials. In several cases, providers have granted permission to send recruitment letters directly to their eligible patients. Programs that serve supportive care needs at the center are also asked to include study information in their newsletters, emails, or social media posts. Following similar procedures, recruitment information and materials have been shared with providers at several other cancer centers in the United States. Outside of cancer centers, these materials are also posted on research portals, posted on social media sites (ie, Facebook, Instagram, Reddit), and provided to patient advocacy and support groups (regionally and nationally) for distribution to their members.

### Procedures

#### Screening and Scheduling

YACS who contact the study team are provided with information about the study including the study information sheet, which provides details of the study aims and procedures. Questions about the study are answered through email or telephone contact. Survivors who are interested in participating are offered the opportunity to schedule a screening meeting via videoconference. In this meeting, survivors can ask any additional questions and complete the eligibility screening, which is conducted by a research assistant. Eligible YACS are then invited to schedule an intervention session, while ineligible survivors are provided with resources [47] for finding help for insomnia. Any questions or concerns about eligibility are referred to the study’s principal investigator (CJR) for clarification and eligibility determination.

#### Intervention Session

**Figure 1:**
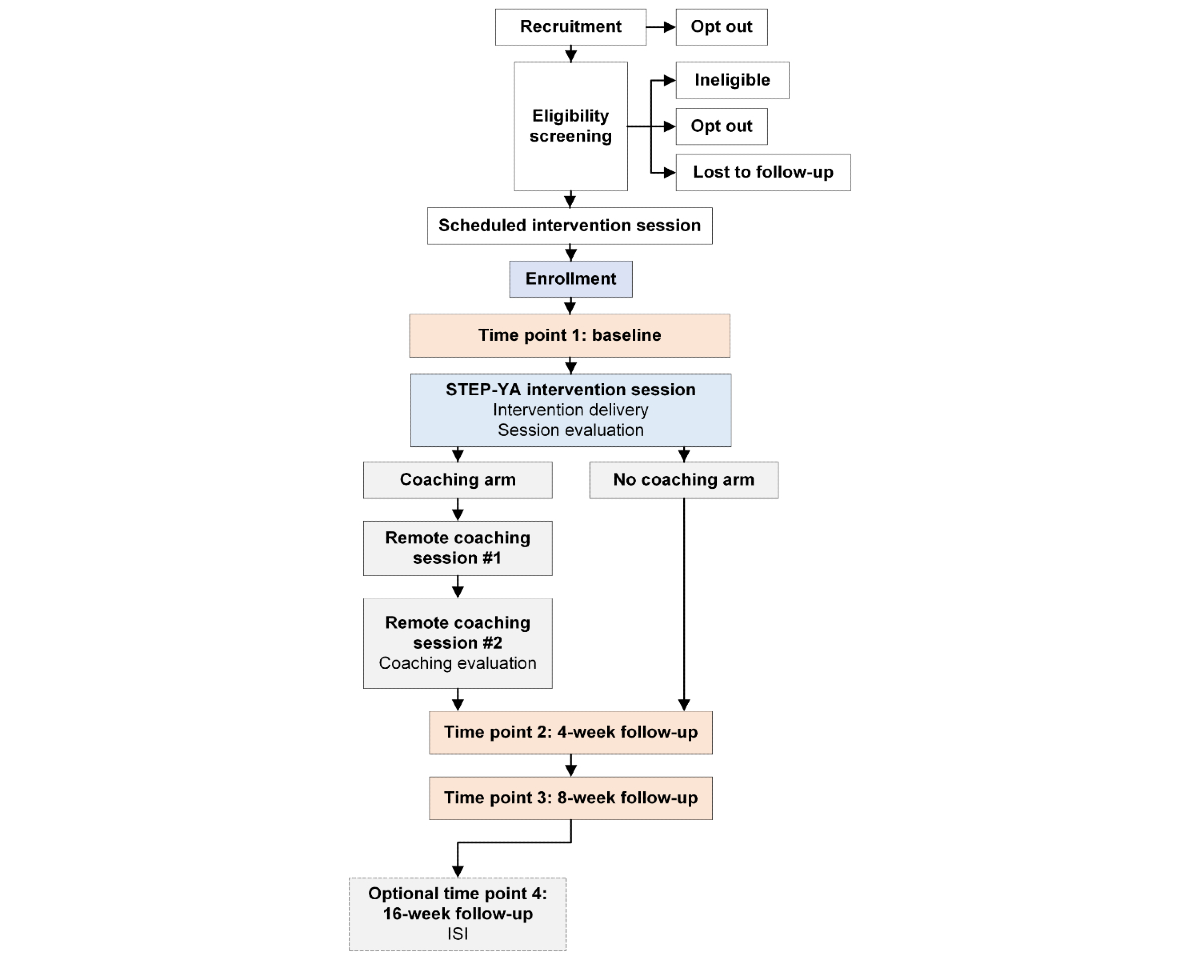
Participant study flow chart for the Sleep Treatment Education Program for Young Adult Cancer Survivors (STEP-YA) study. ISI: Insomnia Severity Index.

In the intervention session, eligible participants complete the consent procedure and a baseline survey before receiving the STEP-YA intervention (Figure 1). Following the intervention, participants are randomized to either the coaching or noncoaching condition, and follow-up contacts are scheduled.

#### Consent Process

YACS who are screened for eligibility more than 2 weeks prior are rescreened for eligibility. Potential participants are provided with a study information sheet, which contains all the elements of informed consent. The interventionist reviews this study information and answers any questions the potential participant may have. Individuals who wish to enroll in the study are then asked to provide verbal consent to the interventionist, who documents this in the study record. This study has been approved for a Waiver of Documentation of Informed Consent, as it is a minimal risk study involving no procedures for which signed consent is generally required outside of the research context.

#### Baseline Survey

After consenting, participants complete a baseline survey. Study staff guide participants in completing the survey using the Qualtrics web-based survey platform [48]. Participants enter data directly into the system, and the study staff do not collect data from participants.

#### STEP-YA Intervention Delivery

STEP-YA is delivered in a single, 90-minute synchronous videoconference session using HIPAA-compliant Zoom technology [49-51] licensed to our cancer center. Interventionists with graduate-level training in psychology, social work, or a closely aligned discipline are trained and supervised by psychologists with CBTI expertise to deliver the STEP-YA intervention. The interventionist delivers STEP-YA in a 1-on-1 session using a slide deck of 47 slides and following a structured outline. During the session, the participant can see and hear the presenter, view the presentation slides, and ask questions. To support participants in developing and implementing their sleep action plan, take-home materials including a copy of the presentation slides and the sleep action plan template are provided by email. The presentation begins with educational information on the problem of insomnia after cancer and the 3-P model of insomnia by Speilman and colleagues [52], before presenting specific suggestions for improving sleep in YACS. These suggestions are based on the CBTI components of sleep hygiene, stimulus control, and cognitive restructuring [29,35,53-55]. To aid comprehension, this material is presented in 4 sections addressing (1) lifestyle issues (eg, benefits of exercise and limiting alcohol), (2) sleep environment (eg, importance of a dark, quiet bedroom; avoiding bed for nonsleep activities), (3) sleep timing (eg, regular wake time, sleep ritual), and (4) managing expectations and challenges (eg, sleep worry, physical symptoms, social pressures). For each suggestion, the underlying rationale and potential benefits are presented. Both the look and content of the STEP-YA material are targeted to appeal to YACS. For example, text describing the benefits of exercise is accompanied by photos of young adults engaged in traditional and contemporary exercises, (eg, yoga, spin class), and the description of the effects of caffeine includes photos of products YACS commonly consume (eg, energy drinks, green tea, sodas, coffee drinks). In section 4, examples of the social, developmental, and health challenges that YACS may encounter are noted, along with suggestions for managing them. These include living spaces shared with roommates or parents, social pressures, “fear of missing out,” and a tendency toward a delayed sleep phase common in young adults, as well as physical health symptoms (eg, pain or hot flashes). At the conclusion of each section (eg, lifestyle), the participant is asked to review the suggestions in the section and complete their sleep action plan template with the interventionist to record their current sleep practices and behavioral changes they intend to make. Action plans of this kind are central to self-management education [41] and have been shown to increase successful behavior change [56,57]. To close the session, the presenter offers examples of STEP-YA suggestions implemented in a daily routine and asks the participant to review and revise their sleep action plan, including anticipating challenges and planning strategies to manage them.

#### Randomization and Follow-Up

After receiving the STEP-YA intervention, participants are randomized using Sealed Envelope [58], a commercially available online software application for randomizing patients into clinical trials. Participants are randomized (1:1) to either (1) STEP-YA alone (noncoaching condition) or (2) STEP-YA with the addition of 2 remote coaching sessions (coaching condition). In order to ensure the 2 arms of the study are balanced by age group, randomization, with a block size of 4, is stratified by those aged 20 years to 29 years versus those aged 30 years to 39 years. Participants are informed of whether they have been randomized to receive the coaching sessions, then they are scheduled for their next telephone contact with the study (Figure 1). Coaching condition participants are scheduled for the first coaching session, and noncoaching participants are scheduled for their 4-week follow-up call. Additionally, participants are asked to provide preferences for all future study contact and correspondence (eg, phone, email, text, mail).

#### Baseline Session Evaluation

At the end of the intervention session, all participants are asked to complete a satisfaction form evaluating their experience with the intervention session. Participants receive a link to a questionnaire on which they report on ease of use and acceptability, satisfaction, and credibility of the session. To promote completion of the questionnaire, participants are encouraged to complete it while still in the intervention session but can choose to complete it later. Participants receive a US $10 gift card upon completion of this evaluation.

#### Postintervention Follow-Up

##### Coaching Sessions

Participants randomized to the coaching condition receive 2 individualized remote coaching sessions conducted via phone call: 1 a week following the STEP-YA session and again 2 weeks following their STEP-YA session. Optimal timing for coaching sessions is 7 days and 14 days after the STEP-YA session but may be scheduled between 5 days and 23 days after the STEP-YA session with at least 5 days between coaching sessions. Paraprofessional coaches (ie, research assistants) trained and supervised by psychologists with CBTI expertise follow a structured coaching outline. In the sessions, designed to last approximately 30 minutes, coaches ask participants to review their progress and challenges implementing their personal sleep action plan. Coaches offer support and encouragement and may refer participants to the take-home materials to help answer questions and reinforce program goals and suggestions. Following principles of self-care management and previous coaching interventions successful with cancer survivors [59-63], coaching sessions are not prescriptive but support YACS to use self-management skills, including decision-making, goal setting, self-assessment, and problem-solving, to help them implement their individual sleep action plan. Administrative data on coaching session duration and sessions missed, interrupted, or rescheduled are collected.

##### Outcome Data Collection

At 4 weeks and 8 weeks postbaseline, study staff recontact participants by telephone at previously scheduled times, ask them to enter follow-up data directly into Qualtrics, and provide technical assistance if needed. Participants who cannot complete the full assessment at that time due to time constraints are asked to complete the 7-item ISI (primary outcome measure) verbally, and their responses are collected; they are then asked to complete the full questionnaire at their earliest convenience. Participants receive emailed reminders in advance of each phone call. At the 4-week call, participants schedule a phone call for the 8-week assessment. Upon completion of the 4 and 8-week assessments, participants receive a US $25 gift card. At the time of their 8-week follow-up phone call, participants are asked if they would be willing to complete an optional assessment 2 months later. Participants who agree receive a Qualtrics link to the 7-item ISI 16 weeks after the intervention session by email. This takes approximately 1 minute to 2 minutes to complete, and participants do not receive an incentive for this optional assessment. The link is sent again 1 week later for nonresponders, but participants receive no additional reminders.

#### Intervention Training and Supervision

##### STEP-YA Intervention

Interventionist trainees familiarize themselves with the STEP-YA protocol and participant-facing STEP-YA materials and discuss these materials with the principal investigator. They are asked to closely review the STEP-YA presentation slides and the structured outline detailing how each slide should be presented to participants before reviewing audiotaped, videotaped, and transcribed STEP-YA sessions. When possible, and with the consent of a participant, trainees attend at least 1 live intervention session to observe STEP-YA administration. Following the session, the interventionist answers any questions the trainee might have and demonstrates postsession procedures. After completing these steps, trainees deliver the STEP-YA intervention in mock interviews with a research assistant (≥2 interviews) and an experienced interventionist (≥1 interview) playing the role of a study participant. Mock sessions are transcribed, scored for fidelity, and reviewed by the principal investigator. Interventionists are approved to conduct STEP-YA sessions with study participants after reliably demonstrating the ability to follow the structured outline for each slide of the STEP-YA presentation in a final mock interview. All interventionists record and transcribe an intervention session (recording only themselves, not the participant) after every 5 to 10 participants. These transcriptions are scored for fidelity and discussed with the principal investigator to ensure adherence to the STEP-YA intervention outline and to reduce deviation between interventionists.

##### Coaching

Paraprofessionals are trained as STEP-YA coaches using a coaching session outline and semistructured script. Coach trainees listen to a minimum of 3 live coaching sessions conducted by an experienced coach and discuss the flow of the script based on participants’ reports of sleep action plan challenges and successes. Trainees then conduct 2 audiotaped mock coaching sessions with an experienced coach or psychologist with CBTI experience acting as a participant and receive feedback on the content of the sessions. Finally, trainees conduct at least 2 live sessions with study participants accompanied in the session by an experienced coach serving as a session co-leader. With approval of the principal investigator (CJR), coaches are then able to conduct their own sessions with study participants.

### Measures

Outcomes for the trial are change in insomnia symptoms as measured by the ISI (primary) and change in mood symptoms as measured by the Profile of Mood States Short form (POMS-SF; secondary) [64]. In addition to these scales, measures of demographic and health variables are included, as are measures of participant experience and satisfaction with the intervention. Details of all measures and time points they are given are included in Table 2.

**Table 2:** Study measures.

Measure	Baseline	Coaching session #2	4-week follow-up	8-week follow-up	Optional 16-week follow-up
Demographic information	X	—^a^	—	—	—
Medical information	X	—	—	—	—
Morningness-Eveningness Questionnaire (MEQ)	X	—	—	—	—
SF-12 Item: Health Rating	X	—	X	X	—
Pain thermometer	X	—	X	X	—
Insomnia Severity Index (ISI)	X	—	X	X	X
Profile of Mood States: Short Form (POMS-SF)	X	—	X	X	—
Session evaluation	X	—	—	—	—
Telehealth Usability Questionnaire	X	—	—	—	—
Coaching evaluation	—	X	—	—	—
Sleep treatment change	—	—	X	X	—

^a^Not measured at this time point.

### Statistical Analysis

#### Endpoints

This is a randomized 2-arm trial evaluating the STEP-YA intervention when delivered with and without a supplementary remote coaching session. The primary endpoint is the change in ISI score from baseline to 8 weeks postintervention. The secondary endpoint is the change in mood symptoms on the POMS-SF from baseline to 8 weeks postintervention. Change scores will be treated as continuous variables, and primary analyses will use analysis of variance methods. Cohen *d* will be used to quantify within-group effect sizes, and Hedges *g* adjustment will estimate between-group effect sizes. Data collected at the 4-week postintervention assessment will be analyzed using similar methods for descriptive and exploratory purposes.

#### Missing Data

Participants are randomized after completing a baseline assessment so complete baseline data are assured. Primary analysis will be an intent-to-treat analysis of randomized participants. Based on experience using similar data collection methods, we anticipate minimal missing data (<10%). If needed, we will use multiple imputation to account for missing data. Potential factors in the imputation model include baseline sleep and mood variables. Sensitivity analysis will be used to evaluate the potential impact of imputation on results.

## Results

### Primary and Secondary Outcomes

Aim 1 of the study will be addressed using a within-subjects analysis including all participants to determine if the STEP-YA intervention is associated with significant changes in outcome measures (insomnia and mood) over time. Aim 2 will be addressed using 2-way analysis of variance with 1 between-subjects factor (coaching vs noncoaching) and 1 within-subjects factor (time) and an interaction term.

To address the secondary exploratory aim, logistic regression analysis will be used to identify participant factors associated with a clinically significant response to the STEP-YA intervention. Analyses will attempt to identify demographic and sleep factors (eg, age, severity of insomnia symptoms) associated with a reduction of ISI scores ≥6 points at 8 weeks. Variables identified in univariate analysis (*P*<.10) will be entered into a multivariable model estimating STEP-YA response from baseline variables. Analyses will explore these relationships separately in the 2 study arms; pooled analyses including participants across arms will be conducted if univariate analyses indicate the relationships of predictors and outcome are consistent.

### Sample Size and Statistical Power for the Primary Outcome

To meet study aims, we require 64 participants with evaluable data but will enroll 74 as a hedge against attrition. Our prior single-session educational interventions have demonstrated large effects on insomnia symptoms of *d*≥1.00; the study is conservatively powered to detect an effect size of .52 (primary aim). With sample size of 64 (32 per arm), the study will have 80% power to detect an effect of this size within each arm (α=.05). To compare the relative improvement in the primary endpoint between arms, 64 subjects will have 80% power to detect a difference of *d*=0.62 between arms (α=.05, 1-sided).

### Timeline

Enrollment began in November 2022. As of September 2023, 30 participants had been enrolled. We anticipate recruitment will be completed in 2024.

## Discussion

### Overview

STEP-YA was developed to meet YACS’ need for effective behavioral insomnia treatment by addressing the low completion rates and lack of access to care common to standard CBTI treatment [30,37,65] and by targeting developmental, social, and health factors that affect their sleep. As a low-intensity intervention, STEP-YA is not intended to completely replace intensive CBTI treatment, which some YACS likely require. Rather, by meeting the needs of the majority of YACS for whom standard insomnia treatments are not appealing, not needed, or currently not available, STEP-YA aims to reduce treatment barriers and avoid potential overtreatment. Though adapted from 2 previously tested brief behavioral interventions for insomnia with cancer survivors [40,66], STEP-YA is a novel intervention as yet unsupported by clinical testing. As recommended in the ORBIT model for development and testing of novel behavioral interventions [42-44], this trial will provide the first empirical evidence evaluating the efficacy of STEP-YA. Study results will help determine whether evidence of efficacy is sufficient to warrant future trials of the existing STEP-YA intervention and aid in the design of future trials investigating its clinical effectiveness in clinical practice. Additionally, results will help to identify subgroups of YACS most likely to benefit from STEP-YA and help to determine whether individualized coaching or other efforts to further optimize the intervention are warranted. We anticipate that data supporting the efficacy of STEP-YA would lead to Phase 3 efficacy trials of STEP-YA in larger and more diverse populations. In addition, positive trial results would also support implementation research into the potential to deliver STEP-YA in small groups and in collaboration with existing survivor advocacy and support groups, which could increase access to insomnia treatment for YACS.

### Limitations and Strengths

Conceptualized within the ORBIT model, this early (stage 2a) trial is designed with a limited scope; it is not intended to conclusively evaluate the efficacy of STEP-YA but to determine if preliminary evidence supporting “proof of concept” for STEP-YA is sufficient to warrant continued efficacy research. As such, the trial design has several notable limitations. Without a control group, study results cannot evaluate how much the observed trial effects are due to natural changes over time or nonspecific effects associated with trial participation. With a modest sample size of English-speaking participants, data on how demographic, cultural, and other participant variables moderate the effects of the intervention will be limited. Similarly, the study will not be able to shed light on any long-term effects of the intervention for participants beyond the 8 and 16-week time points.

Despite these limitations, the trial is well-positioned to provide new and clinically relevant information regarding insomnia interventions for YACS. STEP-YA represents a significant innovation in behavioral insomnia treatment for YACS by delivering a self-management intervention that is less demanding than standard CBTI and attends to YACS specific concerns. In addition, by using nonclinician interventionists to deliver the intervention synchronously online, it aims to overcome logistical barriers and increase access while avoiding uptake and adherence challenges common to asynchronous insomnia interventions [38,67-70]. Investigating the efficacy of this approach is critical for evaluating the potential utility of the STEP-YA intervention and for the potential of applying these intervention methods to address other behavioral needs in this vulnerable population.

### Conclusion

It is well known that behavioral treatment significantly reduces insomnia symptoms but only for those who can access it, which most YACS cannot [30,31]. Our work aims to provide YACS access to empirically supported behavioral insomnia treatment by developing and testing a brief, low-intensity intervention that addresses their needs and interests and can be delivered to them wherever they have internet access. If shown to be effective, this approach has the potential to greatly improve access to the growing population of YACS whose health and quality-of-life are affected by insomnia.

## Appendix

Multimedia Appendix 1 Funding information.

## Abbreviations

CBTI: cognitive behavioral therapy for insomnia
ISI: Insomnia Severity Index
ORBIT: Obesity-Related Behavioral Intervention Trials
POMS-SF: Profile of Mood States: Short Form
STEP-YA: Sleep Treatment Education Program for Young Adult Cancer Survivors
YACS: young adult cancer survivors

## Data Availability

The data sets generated and analyzed during this trial will be available from the corresponding author on reasonable request following completion of the trial and publication of the main outcomes.
