# Supplementary material for: Sleep Treatment Education Program for Young Adult Cancer Survivors (STEP-YA): Protocol for an Efficacy Trial
Source: JMIR Res Protoc. 2023 Nov 29;12:e52315. doi: 10.2196/52315 (PMC10719817; doi:10.2196/52315)
Supplement: Multimedia Appendix 1 [file resprot_v12i1e52315_app1.pdf]

SUMMARY STATEMENT

(Privileged Communication)

PROGRAM CONTACT:
Michelle Mollica
240-276-7621
michelle.mollica@nih.gov

Release Date: 12/14/2021
Revised Date:

Application Number: 1 R21 CA261863-01A1
Formerly: 1R21CA261863-01

Principal Investigator

RECKLITIS, CHRISTOPHER JOHN

Applicant Organization: DANA-FARBER CANCER INST

Review Group: ZCA1 SRB-T (J1)
National Cancer Institute Special Emphasis Panel
Pediatric and AYA Cancer Survivors (R01 & R21)

Meeting Date: 12/01/2021
Council: JAN 2022
Requested Start: 04/01/2022
RFA/PA: CA20-028
PCC: MMOR

Project Title: STEP-YA: An Online Self-Management Intervention for Young Adult Cancer Survivors with Insomnia
SRG Action: Impact Score:25
Next Steps: Visit https://grants.nih.gov/grants/next_steps.htm
Human Subjects: 30-Human subjects involved - Certified, no SRG concerns
Animal Subjects: 10-No live vertebrate animals involved for competing appl.
Gender: 1A-Both genders, scientifically acceptable
Minority: 1A-Minorities and non-minorities, scientifically acceptable
Age: 7A-Only Adults, scientifically acceptable

Project Year	Direct Costs Requested	Estimated Total Cost
1	150,000	267,000
2	125,000	222,500
TOTAL	275,000	489,500

RESUME AND SUMMARY OF DISCUSSION: This is a randomized trial that proposes to test a brief online cognitive behavioral therapy for insomnia (CBTI) intervention and to examine whether two additional coaching sessions are associated with greater benefits. The remotely delivered intervention is innovative in that it is delivered in a single synchronous session, which addresses common barriers to CBTI access and adherence and is highly scalable and also encourages a self-management approach that is developmentally appropriate for YACS. The investigators are experts in CBTI for YACS, and this work is a logical next step for their preliminary work supporting the benefits of online CBTI and single-session sleep interventions for cancer survivors. The environment is outstanding. The resubmission was responsive to reviewers' comments. Among the many strengths, a few minor weaknesses are evident. The pre-post outcomes to be evaluated consist of two self-report scales; additional measures of sleep health or behavior would strengthen the application, as would consideration of whether any benefits are sustained beyond 8 weeks. Overall, this application is rated Outstanding.

DESCRIPTION (provided by applicant): Young adult cancer survivors (YACS) are particularly vulnerable to late-effects of cancer therapy including insomnia:1-8 1 in 4 YACS reports significant insomnia even years after completing treatment.5-7, 9 Chronic insomnia is associated with significant health problems (e.g., heart disease, obesity, hypertension, diabetes, depression, and anxiety).10-19 As YACS are vulnerable to many of these same conditions, providing them with effective insomnia treatment is critically important to their health and development.20-22 Cognitive-behavioral therapy for insomnia (CBTI) is a well-established, empirically supported treatment for insomnia. Multiple randomized trials have demonstrated its effectiveness in the general population and in cancer survivors,23-25 26 and professional guidelines endorse CBTI as "front-line treatment" for insomnia.27 Unfortunately, despite compelling evidence, this empirically validated treatment is largely unavailable to the growing population of YACS who need it.28, 29 Barriers include lack of trained providers,30 high burden of conventional CBTI, and lack of CBTI treatments targeting their specific needs. To address these barriers and deliver effective insomnia treatment to YACS, empirically supported CBTI interventions for survivors of adult cancers were used to develop the Sleep Treatment Education Program for Young Adult Cancer Survivors (STEP-YA) as a brief online CBTI intervention. Delivered in a 90-minute, synchronous 1:1 session led by an instructor, STEP-YA explains causes of insomnia, introduces CBTI principles and methods, and addresses developmental, social, and health factors specifically affecting YACS's sleep. Using a symptom self-management approach, STEP-YA supports survivors in implementing program recommendations into their daily lives with guided behavioral planning. Following recommendations for development and early testing of behavioral interventions,31 an optimization trial is proposed to evaluate efficacy of STEP-YA to improve insomnia in YACS. In this trial, 94 off- treatment YACS (ages 20-39) will be randomized (1:1) to receive the STEP-YA intervention either, 1) alone (non-coaching condition), or 2) with the addition of 2 remote coaching sessions (coaching condition). All study activities will be completed remotely. Specific aims are: 1) To test the hypothesis that STEP-YA improves insomnia symptoms (primary outcome) and mood (secondary outcome), and 2) To compare the improvement in these outcomes in the coaching versus non-coaching conditions. A secondary aim will explore participant factors associated with clinically significant response to the intervention. STEP-YA addresses barriers to insomnia treatment for YACS survivors because it is brief, low cost, low burden, and targets their specific needs. STEP-YA is the first brief CBTI intervention develop specifically for YACS. Evaluating the efficacy of STEP-YA and the utility of individualized coaching in this trial are critical next steps for optimizing the intervention, planning future large scale trials, and ultimately insuring that STEP-YA is effective and widely available to improve the health and quality-of-life of YACS suffering from chronic insomnia.

PUBLIC HEALTH RELEVANCE: As many as 1 in 4 young adult cancer survivors suffer from chronic insomnia, yet evidence-based behavioral treatments to address their insomnia remain largely

unavailable. To address this, we propose to test STEP-YA, an online educational intervention delivered by a trained presenter. By reducing the barriers to evidence-based treatment, STEP-YA has the potential to make effective insomnia treatment widely available for young adult cancer survivors, significantly improving their health and quality of life.

CRITIQUE: The written critiques of individual reviewers are provided in essentially unedited form in this section. Please note that critiques and criteria scores, prepared prior to the review meeting, may not have been revised following discussions at the meeting. The "Resume and Summary of Discussion" section summarizes the final opinions of the review committee.

CRITIQUE 1

Significance: 3
Investigator(s): 1
Innovation: 2
Approach: 4
Environment: 1

Overall Impact
This is a randomized trial that proposes to test a brief online cognitive behavioral therapy for insomnia (CBTI) intervention and to examine whether two additional coaching sessions are associated with greater benefits. The remotely-delivered intervention is innovative in that it is delivered in a single synchronous session, which addresses common barriers to CBTI access and adherence and is highly scalable and also encourages a self-management approach that is developmentally appropriate for YACS. The investigators are experts in CBTI for YACS, and this work is a logical next step for their R03 and other preliminary work supporting the benefits of online CBTI and single-session sleep interventions for cancer survivors. The environment is outstanding. The strength of the preliminary data is somewhat limited in that only pre-post change in self-report measures resulting from single-arm trials are presented. The comparison condition, which is the single-session intervention with two additional coaching sessions, is not well-justified scientifically and the lack of a control arm remains a concern. The pre-post outcomes to be evaluated consist of two self-report scales; additional measures of sleep health or behavior would strengthen the rigor of this proposal, as would consideration of whether any benefits are sustained beyond 8 weeks.

1. Significance
Strengths
- 1 in 4 YACS experience insomnia after cancer treatment, but insomnia often remains unaddressed
- CBTI is effective at improving insomnia in cancer survivors but not accessible to most survivors
- STEP-YA is brief (single session), delivered online, and tailored to meet some developmental needs (such as delayed sleep phase, hot flashes and other symptoms, and autonomy goals)
- Remotely-delivered single session is highly scalable and time- and cost-efficient

Weaknesses
- Clinical significance of pre-post changes in ISI or POMS and implications of these changes for sustained YACS health and quality of life not clear

2. Investigator(s)
Strengths

- PI Recklitis is a clinical psychologist with experience delivering CBT interventions to AYA cancer survivors, including CBTI
- Co-I Zhou is a clinical psychologist and behavioral sleep medicine expert and has published on related work with Recklitis
- Co-I London is a biostatistician with focused expertise in clinical trials with children and AYA cancer and blood disorder patients

Weaknesses
- None noted.

3. Innovation
Strengths
- Single-session intervention is innovative and tests the limits of brief behavioral interventions
- Remote delivery increases scalability
- Addressing developmental needs, including need for autonomy through self-management, is novel

Weaknesses
- Intervention materials could be more interactive to support continued self-management
- Additional measures of sleep health or behavior would increase the rigor of the proposal

4. Approach
Strengths
- Project evaluates effects of STEP-YA (with and without coaching) on insomnia symptoms and mood in YACS with insomnia
- Preliminary R03-funded work supports the efficacy of 3-session CBTI for cancer survivors as well as a single-session educational session, the feasibility of online delivery of CBTI, and benefits of a single session CBTI intervention
- The intervention is well-described, uses materials that have been reviewed and positively rated by YACS, and a logical extension of the team's previous work
- Remote delivery and single session addresses common barriers to CBTI access/adherence

Weaknesses
- Rationale for behavioral coaching and for focusing on the incremental benefits of this intervention component unclear and not well-supported by preliminary work
- No control condition
- Preliminary work describes pre-post changes from single-arm trials only
- No objective measures of sleep behavior, which would be less subject to pre-post demand characteristics than the ISI
- Assessment of outcomes at 4- and 8-weeks post-baseline only, limiting ability to evaluate whether any benefits are sustained

5. Environment
Strengths
- DFCI has outstanding resources to support the proposed research

Weaknesses
- None noted.

Study Timeline
Strengths
- Reasonable

Weaknesses

- None noted.

Protections for Human Subjects
Acceptable Risks and/or Adequate Protections
Data and Safety Monitoring Plan (Applicable for Clinical Trials Only):
Acceptable

Inclusion Plans
- Sex/Gender: Distribution justified scientifically
- Race/Ethnicity: Distribution justified scientifically
- For NIH-Defined Phase III trials, Plans for valid design and analysis: Scientifically acceptable
- Inclusion/Exclusion Based on Age: Distribution justified scientifically

Vertebrate Animals
Not Applicable (No Vertebrate Animals)

Biohazards
Not Applicable (No Biohazards)

Resubmission
- Investigators were moderately responsive to previous reviews

Select Agents
Not Applicable (No Select Agents)

Resource Sharing Plans
Acceptable

Authentication of Key Biological and/or Chemical Resources
Not Applicable (No Relevant Resources)

Budget and Period of Support
Recommend as Requested

CRITIQUE 2

Significance: 1
Investigator(s): 1
Innovation: 2
Approach: 2
Environment: 1

Overall Impact
This is a high impact proposal with relatively minor weaknesses. The investigators are expert in delivering insomnia treatment to AYA survivors of cancer and propose to evaluate the efficacy of their pilot work (funded by a R03 award) in a moderately scaled R21 that follows the optimization framework laid out in the ORBIT model. The proposal of a relatively simple, but powerful, intervention within a videoconference medium allows the investigators to address an initial efficacy question within the R21 budget. This is innovative and represents a good return on investment. Some tradeoffs are necessary

with this strategy, however. Impact is diminished by lack of attention to the daily sleep data, and choice not to include objective assessment of sleep in even a subsample of participants.

1. Significance
Strengths
- Insomnia is a significant problem in AYA cancer survivors. Moreover, techniques for improving insomnia are well-researched and understood.
- The team has a compelling intervention ready to deploy to AYAs with insomnia and the prior publications point logically toward this application as a next step.
- The introduction to the application argues that this approach is different from other currently available techniques in its brevity.
- Couching this work in the context of the ORBIT model and using this R21 to optimize the intervention is a strength that adds impact.

Weaknesses
- None noted.

2. Investigator(s)
Strengths
- The team is excellent and obviously capable of the work based on their prior publications and pilot data.

Weaknesses
- None noted.

3. Innovation
Strengths
- Tailoring the treatment to the needs of the patient and using that tailoring to decrease the time and effort in intervention is innovative.
- The remote delivery of this single session intervention is innovative.

Weaknesses
- None noted.

4. Approach
Strengths
- The team has translated evidence-based strategies for insomnia into an intervention that is ready for deployment.
- The use of the RCT is a strength as it allows the team to prepare for future efficacy work.
- The strategies in the intervention are appropriate and evidence-based.
- The intervention is brief which will help with uptake and should yield high engagement.
- There is evidence that the target outcomes are modifiable.
- The standard of care is appropriate.
- Inclusion and exclusion is appropriate.

Weaknesses
- It is within the team’s capability to objectively assess sleep. However, in this resubmission that suggestion was not taken. Self-reported outcomes have low rigor. Accelerometers combined with a daily sleep log are a good middle ground.
- Sleep is a daily variable and there is a missed opportunity to capture the time-series data.

5. Environment
Strengths

- The environment is excellent.

Weaknesses
- None noted.

Study Timeline
Strengths
- The timeline is appropriate for the aims and feasible.

Weaknesses
- None noted.

Protections for Human Subjects
Acceptable Risks and/or Adequate Protections
- The plan is detailed and appropriate.

Data and Safety Monitoring Plan (Applicable for Clinical Trials Only):
Acceptable
- o Data safety and HIPAA compliance are described, as are encryption protocols.

Inclusion Plans
- Sex/Gender: Distribution justified scientifically
- Race/Ethnicity: Distribution justified scientifically
- Inclusion/Exclusion Based on Age: Distribution justified scientifically
- Justified scientifically.

Vertebrate Animals
Not Applicable (No Vertebrate Animals)

Biohazards
Not Applicable (No Biohazards)

Resubmission
- The introduction to the application is well-detailed and the team has made a number of important changes that increase impact. Some notable weaknesses in the methodology remain, and the team attempts to justify them with argumentation. It seems that even if there are limits to including a technique like accelerometers, the benefits of including them in even a subset of participants should be clear to the team and could significantly enhance their preparedness for the full RCT.

Select Agents
Not Applicable (No Select Agents)

Resource Sharing Plans
Acceptable

Authentication of Key Biological and/or Chemical Resources
Not Applicable (No Relevant Resources)

Budget and Period of Support
Recommend as Requested

CRITIQUE 3

Significance: 2
Investigator(s): 1
Innovation: 2
Approach: 3
Environment: 1

Overall Impact
Insomnia often becomes chronic, affecting ~ 25% of YACS. No evidence-based treatments available for YACS. The investigators propose optimization of a pilot tested brief online educational intervention (based on CBT-I, and self-management principles), targeting insomnia specifically in YACS. The PI proposes to test the hypothesis that STEP-YA improves insomnia symptoms (primary outcome) and mood (secondary outcome) and compare coaching versus non-coaching conditions. A secondary aim will explore participant factors associated with clinically significant response to the intervention. The resubmission was responsive to reviewers' comments. The strength of the proposal is the significance of the problem; the innovative intervention, primary outcome, sample size was based on pilot studies targeting the needs of YACS. Weaknesses that moderate the impact included lack of time control for the coaching control arm; An additional control arm may be needed to eliminate the placebo effect. Lack of objective measures to assess insomnia such as actigraphy may be a limitation.

1. Significance
Strengths
- Insomnia often becomes chronic, affecting as many as 25% of YACS.
- Insomnia increases the risk for not achieving educational, social, and vocational milestones critical to young adult development.
- CBT-I is a gold standard for treatment of insomnia in adult cancer patients is often not often available and doesn't target mechanisms that cause insomnia in YACS.
- The investigators propose optimization of an online educational intervention targeting insomnia in YACS.
- Responsive to RFA

Weaknesses
- None noted.

2. Investigator(s)
Strengths
- The PI and co- investigators have prior collaboration between them and have expertise to complete the study.

Weaknesses
- None noted.

3. Innovation
Strengths
- Optimization of an online educational intervention targeting insomnia in YACS.
- Intervention developed on ORBIT model.
- Brief intervention (single synchronous online session) and self-management approach

Weaknesses
- None noted.

4. Approach

Strengths
- Intervention based on pilot data.
- Randomized control trial design.
- Eligibility criteria of presence of ISI>/=12.
- Primary outcome measure (ISI), and sample size based on preliminary study.
- Inclusion of minority participants.

Weaknesses
- Lack of time control for the coaching control arm.
- An additional control arm may be needed to eliminate the placebo effect.
- Lack of objective measures to assess the effect of intervention on insomnia such as actigraphy may be a limitation.

5. Environment
Strengths
- The environment is conducive to successfully complete the aims outlined in the proposal.

Weaknesses
- None noted.

Study Timeline
Strengths
- Timeline is appropriate to complete the study.

Weaknesses
- None noted.

Protections for Human Subjects
Acceptable Risks and/or Adequate Protections
Data and Safety Monitoring Plan (Applicable for Clinical Trials Only):
Acceptable

Inclusion Plans
- Sex/Gender: Distribution justified scientifically
- Race/Ethnicity: Distribution justified scientifically
- For NIH-Defined Phase III trials, Plans for valid design and analysis: Not applicable
- Inclusion/Exclusion Based on Age: Distribution justified scientifically

Vertebrate Animals
Not Applicable (No Vertebrate Animals)

Biohazards
Not Applicable (No Biohazards)

Resubmission
- The resubmission was responsive to the reviewers' comments.

Select Agents
Not Applicable (No Select Agents)

Resource Sharing Plans
Not Applicable (No Relevant Resources)

Authentication of Key Biological and/or Chemical Resources
Not Applicable (No Relevant Resources)

Budget and Period of Support
Recommend as Requested

THE FOLLOWING SECTIONS WERE PREPARED BY THE SCIENTIFIC REVIEW OFFICER TO SUMMARIZE THE OUTCOME OF DISCUSSIONS OF THE REVIEW COMMITTEE, OR REVIEWERS' WRITTEN CRITIQUES, ON THE FOLLOWING ISSUES:

PROTECTION OF HUMAN SUBJECTS: ACCEPTABLE

INCLUSION OF WOMEN PLAN: ACCEPTABLE

INCLUSION OF MINORITIES PLAN: ACCEPTABLE

INCLUSION ACROSS THE LIFESPAN: ACCEPTABLE

COMMITTEE BUDGET RECOMMENDATIONS: The budget was recommended as requested.

Footnotes for 1 R21 CA261863-01A1; PI Name: Recklitis, Christopher John

NIH has modified its policy regarding the receipt of resubmissions (amended applications).See Guide Notice NOT-OD-18-197 at https://grants.nih.gov/grants/guide/notice-files/NOT-OD-18-197.html. The impact/priority score is calculated after discussion of an application by averaging the overall scores (1-9) given by all voting reviewers on the committee and multiplying by 10. The criterion scores are submitted prior to the meeting by the individual reviewers assigned to an application, and are not discussed specifically at the review meeting or calculated into the overall impact score. Some applications also receive a percentile ranking. For details on the review process, see http://grants.nih.gov/grants/peer_review_process.htm#scoring.

MEETING ROSTER

National Cancer Institute Special Emphasis Panel
NATIONAL CANCER INSTITUTE
Pediatric and AYA Cancer Survivors (R01 & R21)

ZCA1 SRB-T (J1)
12/01/2021 - 12/02/2021

Notice of NIH Policy to All Applicants: Meeting rosters are provided for information purposes only. Applicant investigators and institutional officials must not communicate directly with study section members about an application before or after the review. Failure to observe this policy will create a serious breach of integrity in the peer review process, and may lead to actions outlined in NOT-OD-14-073 at https://grants.nih.gov/grants/guide/notice-files/NOT-OD-14-073.html, NOT-OD-15-106 at https://grants.nih.gov/grants/guide/notice-files/NOT-OD-15-106.html, and NOT-OD-18-115 at https://grants.nih.gov/grants/guide/notice-files/NOT-OD-18-115.html, including removal of the application from immediate review.

CHAIRPERSON(S)

FRIEDMAN, DEBRA L, RN, MD
PROFESSOR
DIVISION OF PEDIATRIC ONCOLOGY
AND HEMATOLOGY
E. BRONSON INGRAM CHAIR AND DIRECTOR
VANDERBILT UNIVERSITY
NASHVILLE, TN 37232

HENDERSON, TARA OLIVE, MD, MPH
PROFESSOR
DEPARTMENT OF PEDIATRIC HEMATOLOGY, ONCOLOGY
AND STEM CELL TRANSPLANTATION
THE UNIVERSITY OF CHICAGO MEDICINE
CHICAGO, IL 60637

PERENTESIS, JOHN PETER, MD
PROFESSOR
DEPARTMENT OF PEDIATRICS
KLEISINGER CHAIR FOR NEW CANCER THERAPIES
CINCINNATI CHILDREN'S HOSPITAL
UNIVERSITY OF CINCINNATI COLLEGE OF MEDICINE
CINCINNATI, OH 45229-3039

MEMBERS

AUNE, GREGORY J., MD, PHD
ASSOCIATE PROFESSOR
DEPARTMENT OF PEDIATRICS HEMATOLOGY/ONCOLOGY
UNIVERSITY OF TEXAS HEALTH SCIENCE CENTER
SAN ANTONIO, TX 78207

BENTZEN, SOREN M, PHD, DSC
PROFESSOR
DEPARTMENT OF EPIDEMIOLOGY AND PUBLIC HEALTH
UNIVERSITY OF MARYLAND
BALTIMORE, MD 21201

BRADLEY, CATHY J, MPA, PHD
PROFESSOR AND ASSOCIATE DIRECTOR
DEPARTMENT OF HEALTH SYSTEMS,
MANAGEMENT AND POLICY
UNIVERSITY OF COLORADO
AURORA, CO 80045

BUCHBINDER, DAVID KYLE, MD
PROFESSOR
DEPARTMENT OF HEMATOLOGY AND PEDIATRICS
UNIVERSITY OF CALIFORNIA AT IRVINE
IRVINE, CA 92697

CAMPBELL, KRISTIN, PHD
ASSOCIATE PROFESSOR
DEPARTMENT OF PHYSICAL THERAPY
CLINICAL EXERCISE PHYSIOLOGY LAB
UNIVERSITY OF BRITISH COLUMBIA
VANCOUVER, BC V6T 1Z3
CANADA

CANTER, KIMBERLY, PHD
CLINICAL PSYCHOLOGIST
DEPARTMENT OF PEDIATRICS
DIVISION OF BEHAVIORAL HEALTH
NEMOURS CHILDREN HEALTH
WILMINGTON, DE 19803

CASILLAS, JACQUELINE, MD
ASSOCIATE PROFESSOR, DEPARMENT OF PEDIATRICS
DEPARTMENT OF PEDIATRICS
UCLA PEDIATRIC CANCER SURVIVORSHIP PROGRAM
DAVID GEFFEN SCHOOL OF MEDICINE
UNIVERSITY OF CALIFORNIA AT LOS ANGELES
LOS ANGELES, CA 90095

COLE, PETER D, MD
PROFESSOR, EMBRACE KIDS FOUNDATION CHAIR
DEPARTMENT OF PEDIATRICS
DIVISION OF PEDIATRIC HEMATOLOGY/ONCOLOGY
ROBERT WOOD JOHNSON MEDICAL SCHOOL
RUTGERS CANCER INSTITUTE OF NEW JERSEY
NEW BRUNSWICK, NJ 08903

CUSHING, CHRISTOPHER C, PHD
ASSISTANT PROFESSOR AND SCIENTIST
DEPARTMENT OF CLINICAL CHILD
PSYCHOLOGY PROGRAM
UNIVERSITY OF KANSAS
LAWRENCE, KS 66045

DORRIS, KATHLEEN, MD
ASSOCIATE PROFESSOR
DEPARTMENT OF PEDIATRICS
CHILDREN'S HOSPITAL COLORADO
UNIVERSITY OF COLORADO
AURORA, CO 80045

DUNCAN, FRANCESCA E., PHD
ASSISTANT PROFESSOR
DEPARTMENT OF OBSTETRICS AND GYNECOLOGY
FEINBERG SCHOOL OF MEDICINE
NORTHWESTERN UNIVERSITY
CHICAGO, IL 60611

GRACIA, CLARISA R., MD
PROFESSOR
DEPARTMENT OF OBSTETRICS AND GYNECOLOGY
PENN FERTILITY CARE
UNIVERSITY OF PENNYSYLVANIA
PHILADELPHIA, PA 19104

HAYES-LATTIN, BRANDON M, MD
PROFESSOR
DIVISION OF HEMATOLOGY AND MEDICAL ONCOLOGY
KNIGHT CANCER INSTITUTE
OREGON HEALTH AND SCIENCE UNIVERSITY
PORTLAND, OR 97239

KEPKA, DEANNA LEE, MPH, PHD
ASSOCIATE PROFESSOR
DEPARTMENT OF NURSING
HUNTSMAN CANCER INSTITUTE
UNIVERSITY OF UTAH
SALT LAKE CITY, UT 84112

LANGER, MARK P., MD
PROFESSOR
DEPARTMENT OF RADIATION ONCOLOGY
CANCER CENTER
INDIANA UNIVERSITY SCHOOL OF MEDICINE
INDIANAPOLIS, IN 46202

LOW, CARISSA A, PHD
ASSISTANT PROFESSOR
DEPARTMENT OF MOBILE SENSING AND
HEALTH INSTITUTE
UPMC HILLMAN CANCER CENTER
UNIVERSITY OF PITTSBURGH
PITTSBURGH, PA 15213

MANNE, SHARON L, PHD
PROFESSOR AND CHIEF
DEPARTMENT OF MEDICINE
CENTER OF EXCELLENCE IN
CANCER SURVIVORSHIP
RUTGERS ROBERT WOOD JOHNSON MEDICAL SCHOOL
NEW BRUNSWICK, NJ 08903

MICHALSKI, JEFF M, MD
PROFESSOR
DEPARTMENT OF RADIOLOGY
MALLINCKRODT INSTITUTE
WASHINGTON UNIVERSITY
ST. LOUIS, MO 63110

MOORE, JUSTIN BRIAN, PHD
ASSOCIATE PROFESSOR
DEPARTMENT OF FAMILY AND COMMUNITY MEDICINE,
EPIDEMIOLOGY AND PREVENTION AND
IMPLEMENTATION SCIENCE
WAKE FOREST SCHOOL OF MEDICINE
WINSTON-SALEM, NC 27157

MUELLER, SABINE, MD, PHD
ASSOCIATE PROFESSOR
DEPARTMENT OF NEUROLOGY
UNIVERSITY OF CALIFORNIA, SAN FRANCISCO
SAN FRANCISCO, CA 94143

PARSONS, HELEN M, MPH, PHD
ASSOCIATE PROFESSOR
DIVISION OF HEALTH POLICY AND MANAGEMENT
SCHOOL OF PUBLIC HEALTH
UNIVERSITY OF MINNESOTA
MINNEAPOLIS, MN 55455

PINTO, BERNARDINE M, PHD
ASSOCIATE DEAN FOR RESEARCH AND PROFESSOR
CO-DIRECTOR CANCER SURVIVORSHIP CENTER
COLLEGE OF NURSING
UNIVERSITY OF SOUTH CAROLINA
COLUMBIA, SC 29208

PIRL, WILLIAM F, MD, MPH
ASSOCIATE PROFESSOR
PSYCHOSOCIAL ONCOLOGY AND PALLIATIVE CARE
DANA-FARBER CANCER INSTITUTE
HARVARD MEDICAL SCHOOL
BOSTON, MA 02115

RATAIN, MARK J, MD
PROFESSOR OF MEDICINE
DEPARTMENT OF MEDICINE
DIVISION OF THE BIOLOGICAL SCIENCES
PRITZKER SCHOOL OF MEDICINE
UNIVERSITY OF CHICAGO
CHICAGO, IL 60637-1463

ROSENBERG, ABBY R, MD
ASSOCIATE PROFESSOR
DEPARTMENT OF PEDIATRICS
PALLIATIVE CARE AND RESILIENCE RESEARCH
SEATTLE CHILDREN'S RESEARCH INSTITUTE
UNIVERSITY OF WASHINGTON SCHOOL OF MEDICINE
SEATTLE, WA 98105

SALZ, TALYA, PHD
RESEARCH SCIENTIST
DEPARTMENT OF EPIDEMIOLOGY AND BIOSTATISTICS
MEMORIAL SLOAN-KETTERING CANCER CENTER
NEW YORK, NY 10065

SHAH, BIJAL DINESH, MD
ASSOCIATE MEMBER
DEPARTMENT OF MALIGNANT HEMATOLOGY
SECTION LEADER, ACUTE LYMPHOBLASTIC
LEUKEMIA AND MANTLE CELL LYMPHOMA
H. LEE MOFFITT CANCER CENTER
TAMPA, FL 33612

SHERWOOD, PAULA R, PHD
PROFESSOR
DEPARTMENT OF ACUTE AND TERTIARY CARE
UNIVERSITY OF PITTSBURGH
PITTSBURGH, PA 15261

SIMPSON, PIPPA M, PHD
PROFESSOR AND DIRECTOR
DIVISION OF QUANTITATIVE HEALTH SCIENCES
MEDICAL COLLEGE OF WISCONSIN
MILWAUKEE, WI 53226

STERN, MARILYN, PHD
PROFESSOR
DEPARTMENT OF CHILD AND FAMILY STUDIES
COLLEGE OF BEHAVIORAL AND COMMUNITY SCIENCES
H. LEE MOFFITT CANCER CENTER
UNIVERSITY OF SOUTH FLORIDA
TAMPA, FL 33612

TAYLOR, JEREMY M.G., PHD
PROFESSOR
DEPARTMENT OF BIOSTATISTICS
PHARMACIA RESEARCH PROFESSOR
UNIVERSITY OF MICHIGAN
ANN ARBOR, MI 48109

VALLE, CARMINA G, MPH, PHD
ASSISTANT PROFESSOR
DEPARTMENT OF NUTRITION
LINEBERGER COMPREHENSIVE
CANCER CENTER
UNIVERSITY OF NORTH CAROLINA, CHAPEL HILL
CHAPEL HILL , NC 27599

WHEELER, STEPHANIE BROOKE, PHD
PROFESSOR
DEPARTMENT OF HEALTH POLICY AND MANAGEMENT
UNIVERSITY OF NORTH CAROLINA AT CHAPEL HILL
CHAPEL HILL, NC 27599-7411

YANEZ, BETINA, PHD
ASSOCIATE PROFESSOR
DEPARTMENT OF PATIENT ENGAGEMENT
AND CANCER SURVIVORSHIP INSTITUTE
ROBERT H. LURIE COMPREHENSIVE CANCER CENTER
NORTHWESTERN UNIVERSITY
CHICAGO, IL 60611

YENNU, SRIRAM, MD
ASSOCIATE PROFESSOR
DEPARTMENT OF PALLIATIVE
DIVISION OF CANCER MEDICINE
REHABILITATION AND INTEGRATIVE MEDICINE
UNIVERSITY OF TEXAS MD ANDERSON CANCER CTR
HOUSTON, TX 77030

SCIENTIFIC REVIEW OFFICER

MEEKER, TIMOTHY C., MD
SCIENTIFIC REVIEW OFFICER
SPECIAL REVIEW BRANCH
DIVISION OF EXTRAMURAL ACTIVITIES
NATIONAL CANCER INSTITUTE
NATIONAL INSTITUTES OF HEALTH
ROCKVILLE, MD 20850

EXTRAMURAL SUPPORT ASSISTANT

GRADINGTON-JONES, IMELA
PROGRAM SPECIALIST
SPECIAL REVIEW BRANCH
DIVISION OF EXTRAMURAL ACTIVITIES
NATIONAL CANCER INSTITUTE SHADY GROVE
NATIONAL INSTITUTES OF HEALTH
BETHESDA, MD 20892-9750

Consultants are required to absent themselves from the room during the review of any application if their presence would constitute or appear to constitute a conflict of interest.
